# GRK2 moderates the acute mitochondrial damage to ionizing radiation exposure by promoting mitochondrial fission/fusion

**DOI:** 10.1038/s41420-018-0028-7

**Published:** 2018-02-14

**Authors:** Antonietta Franco, Daniela Sorriento, Jessica Gambardella, Roberto Pacelli, Nella Prevete, Claudio Procaccini, Giuseppe Matarese, Bruno Trimarco, Guido Iaccarino, Michele Ciccarelli

**Affiliations:** 1 Department of Advanced Biomedical Sciences, “Federico II” University, Naples, Italy; 2 Center for Pharmacogenomics, Washington University in St. Louis, St Louis, USA; 3 Department of Medicine, Surgery and Dentistry, University of Salerno, Baronissi, Italy; 4 Department of Translational Medical Sciences, “Federico II” University, Naples, Italy; 5 Department of Molecular Medicine and Medical Biotechnologies “Federico II” University, Naples and Institute of Experimental Endocrinology and Oncology (IEOS-CNR), Naples, Italy

## Abstract

The modern understanding of the G protein-coupled receptor kinase 2 has grown towards the definition of a stress protein, for its ability to rapidly compartmentalize within the cell in response to acute stimulation. Also, mitochondria can be regulated by GRK2 localization. We show that Ionizing Radiation (IR) exposure acutely damages mitochondria regarding mass, morphology, and respiration, with recovery in a framework of hours. This phenomenon is actively regulated by GRK2, whose overexpression results to be protective, and reciprocally, deletion accelerates degenerative processes. The regulatory effects of the kinase involve a new interactome that includes binding HSP90 and binding and phosphorylation of the key molecules involved in the process of mitochondrial fusion and recovery: MFN-1 and 2.

## Introduction

G-protein coupled receptor kinase 2 (GRK2) regulates multiple cellular functions^1,2^ and is tightly regulated in a time and space-dependent manner by different stimuli or pathophysiological conditions^3,4^. Lately, the evidence that GRK2 can also localize in mitochondria through binding of HSP-90 in response to hypoxia or other stressful events^5,6^, supports the vision of GRK2 as a stress protein, opening a scenario of unexplored paradigms of signaling for the kinase, and prompting further investigation of specific molecular partners in mitochondrial regulation.

Exposure to ionizing radiation (IR) in a daily life, in particular at very low radiation doses, has become very frequent, either for health purposes (screening tests for cancer) or occupational reasons (health professionals, frequent-flyers)^7,8^, and is becoming an issue for public health. Indeed, several studies have elucidated the damages of ionizing irradiation on DNA and the molecular mechanisms that lead to the repair process or cell death. However, accumulating evidence supports an argument of nuclear damage being second in time to the damage of mitochondria^9,10^.

Mitochondrial plasticity represents a sophisticated mechanism of repair and regeneration during which the organelles undergo a process of fission and fusion to remove the dysfunctional mtDNA and damaged respiratory proteins^11,12^. Mitochondrial fission and fusion are highly dynamic and precisely regulated through activation of dynamin-related protein-1 (drp1) and mitofusin 1 and 2 (MFN1 and 2), respectively, to maintain the appropriate number of functional mitochondria and cope with the energetic cellular demand^13–15^.

As above described, GRK2 is emerging as a stress protein, and it has been demonstrated to localize and regulate mitochondria. Thus, in this study, we evaluated the effects of GRK2 subcellular localization in mitochondria of HEK293 upon IR exposure and found that this kinase regulates mitochondrial fusion process through interaction and phosphorylation of MFNs.

## Results

### Acute and transient IR exposure induces mitochondrial structural changes

**Fig. 1:**
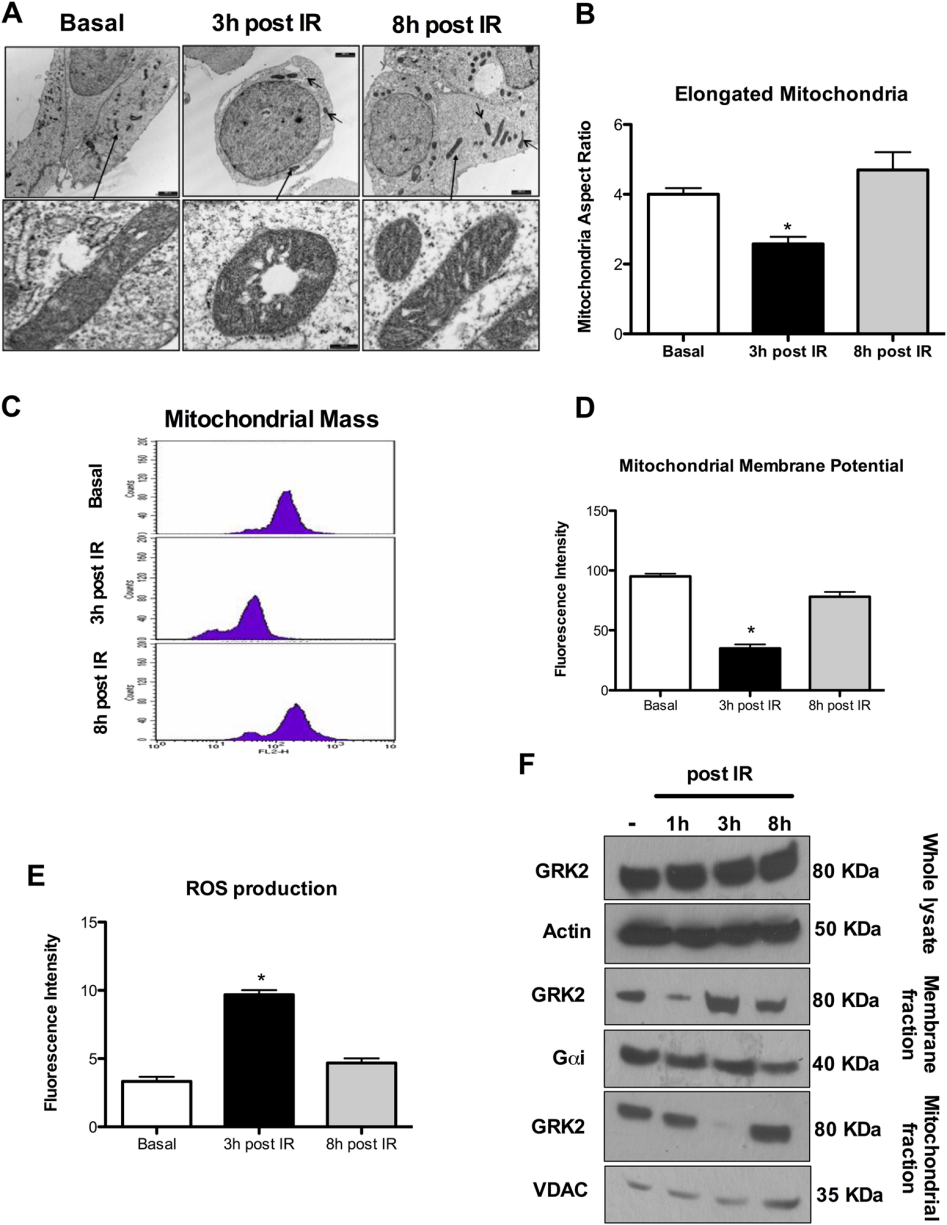
Modifications of mitochondrial morphology, mass and function in HEK293 after IR exposure. **a,** **b** Mitochondrial morphology. Representative TEM images of *n* = 3 independent experiments. The number of elongated mitochondria is reduced along with vacuolization and cristae disarrangement at 3 h post-IR. Mitochondrial morphology and ultrastructure are recovered at 8 h post-IR. **c** Mitochondrial Mass*.* Representative image of flow cytometry of *n* = 5 independent experiments evaluating mitochondrial mass by Mitotracker after IR exposure. IR exposure determinates at 3 h a significant reduction of mitochondrial mass (*p < 0.05 vs. basal) that is recovered at 8 h post-IR. **d** Mitochondrial membrane potential. Mitochondrial membrane potential measured by flow cytometry by TMRE (Mean ± SEM of 5 independent experiments). IR decreases mitochondrial membrane potential at 3 h (*p < 0.05 vs. Basal) with recovering at 8 h post exposure. **e** Mitochondrial ROS production. ROS production measured at flow cytometry by MitoSOX (Mean ± SEM of 5 independent experiments). IR produces mitochondrial ROS accumulation as evident at 3 h (*p < 0.05 vs. Basal) with normalization at 8 h post exposure. **f** GRK2 compartmentalization. GRK2 sub-cellular localization after IR exposure evaluated by cellular fractionation and WB. Representative image of *n* = 3 independent experiments. GRK2 total levels are not significantly modified after IR exposure (Top image; Whole Lysate). At 3 h post-IR, GRK2 mainly localizes at the plasma membrane with the reduction at 8 h post-IR (Middle image; Membrane fraction). GRK2 levels in mitochondria are significantly reduced at 3 h but increased at 8 h post-IR (Bottom image; Mitochondrial fraction)

We evaluated the effects of different doses of IR (1, 2, 4, and 8 Gy) exposure on HEK-293 cells and found a significant mitochondrial recovery after a 4Gy IR exposure (Fig. 1) as assessed by shape, ultrastructure, mass, and function. Analysis of mitochondrial morphology by TEM shows that at 3 h post-IR, the number of elongated mitochondria is significantly reduced with cristae disarrangements and vacuolization as compared to not IR exposed cells; this phenotype is transient and recovered at 8 h post-IR (Fig. 1a, b). Accordingly, mitochondrial mass is reduced at 3 h and retrieved at 8 h post-IR (Fig. 1c). At 3 h post-IR the mitochondria are dysfunctional with reduced membrane potential (Fig. 1d) and inability to counteract ROS accumulation (Fig. 1e). At 8 h post-IR, membrane potential and ROS levels are normalized (Fig. 1d, e).

### Acute and transient IR exposure induces GRK2 subcellular localization

Modification of mitochondrial morphology, mass and function after IR exposure (Fig. 1a–e), is paralleled by modifications in the subcellular localization of GRK2. While total level of GRK2 does not change after IR exposure, GRK2 mainly localizes at plasma membrane at 3 h and preferentially accumulates in mitochondria at 8 h post-IR (Fig. 1f).

### GRK2 removal and overexpression reciprocally modify mitochondrial morphology and function upon IR exposure

**Fig. 2:**
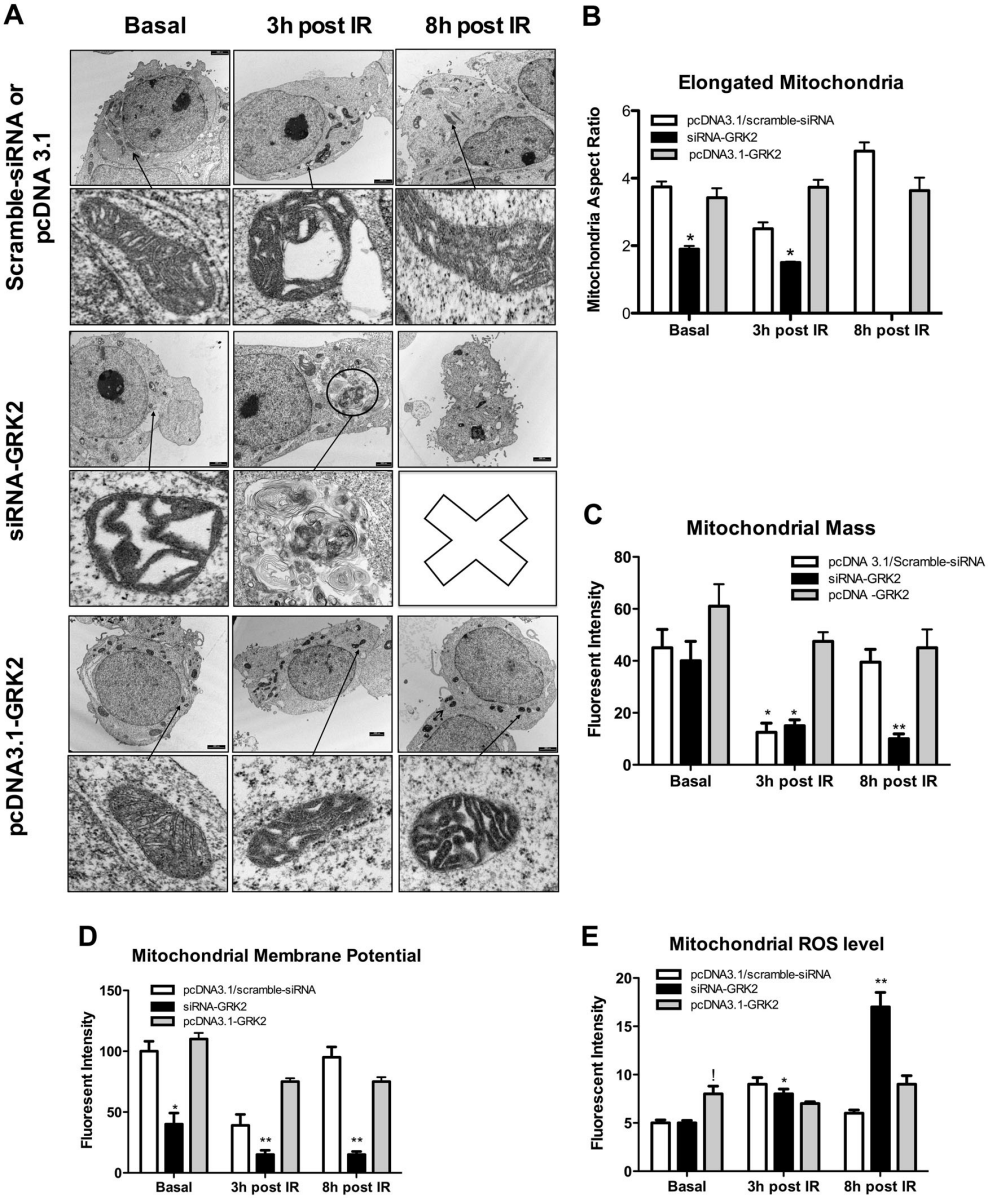
Modification of GRK2 intracellular levels affects mitochondrial morphology and function after IR exposure. **a**, **b** Mitochondrial morphology. Representative TEM images of *n* = 3 independent experiments. As above described, in HEK293 mitochondrial morphology is damaged at 3 h and recovered at 8 h post-IR (Top image; Scramble siRNA, or pcDNA3.1). Silencing of GRK2 by siRNA abolishes mitochondrial recovering. At 3 h post-IR there is the prevalent formation of mitophagosome and reduction of elongated mitochondria. At 8 h post IR, elongated mitochondria cannot be observed (Middle image; siRNA-GRK2). Overexpression of GRK2 by pcDNA3.1 plasmid transfection preserves morphology with a prevalence of elongated mitochondria at 3 h and 8 h post-IR (Bottom image; pcDNA3.1-GRK2). **c** Mitochondrial mass. IR exposure in HEK293 cells produces reduction of mitochondrial mass at 3 h (*p < 0.05 vs. basal) with recovering at 8 post-IR, as above described. In cells treated with siRNA-GRK2, mitochondrial mass is reduced at 3 h (*p < 0.05 vs. basal) and not recovered at 8 h post-IR (!*p* < 0.05 vs. basal). In HEK293 transfected with pcDNA3.1-GRK2 mitochondrial mass is preserved at 3 h and 8 h post-IR. **d** Mitochondrial membrane potential. Mitochondrial membrane potential is reduced at 3 h and recovered at 8 h post-IR in control cells treated with scramble-siRNA. GRK2 silencing in HEK293 reduces membrane potential in basal condition (* vs. scramble-siRNA, *p* < 0.05) with further deterioration at 3 h and 8 h post-IR (** vs. basal, *p* < 0.05). **e** Mitochondrial ROS production. Mitochondrial ROS accumulate at 3 h and normalize at 8 h post-IR in not treated cells. Cells with GRK2 removal show a progressive increase in ROS amount at 3 and 8 h post-IR (*,** vs. basal, *p* < 0.05). Cells with GRK2 overexpression show ROS accumulation in basal condition (! vs. pcDNA3.1; *p* < 0.05) but with no modification at 3 h and 8 h post-IR

To understand whether modifications in subcellular GRK2 localization are epiphenomenal or causative, we assessed GRK2 removal and overexpression on the above-described cellular phenotype. Efficiency of GRK2 overexpression and knock down are shown in Suppl Fig. 1. siRNA-GRK2 treated cells show reduction of elongated mitochondria with ultrastructure alterations already before IR (Fig. 2a, b). At 3 h post-IR mitochondria morphology is further compromised and not recovered at 8 h post IR (Fig. 2a, b). Accordingly, mitochondrial mass progressively decreases through the time (Fig. 2c). At 3 h post-IR mitochondrial function deteriorates with a reduction of mitochondrial membrane potential and ROS accumulation (Fig. 2d, e), with no recovery at 8 h post-IR (Fig. 2d, e). In pcDNA3.1-GRK2 transfected cells, mitochondrial morphology and structure are not modified before IR and preserved at 3 h and 8 h post-IR (Fig. 2a, b). Accordingly, mitochondrial mass (Fig. 2c), mitochondrial membrane potential and ROS level are not modified respect to before IR (Fig. 2d, e).

### Mitochondrial respiration after GRK2 removal and overexpression

**Fig. 3:**
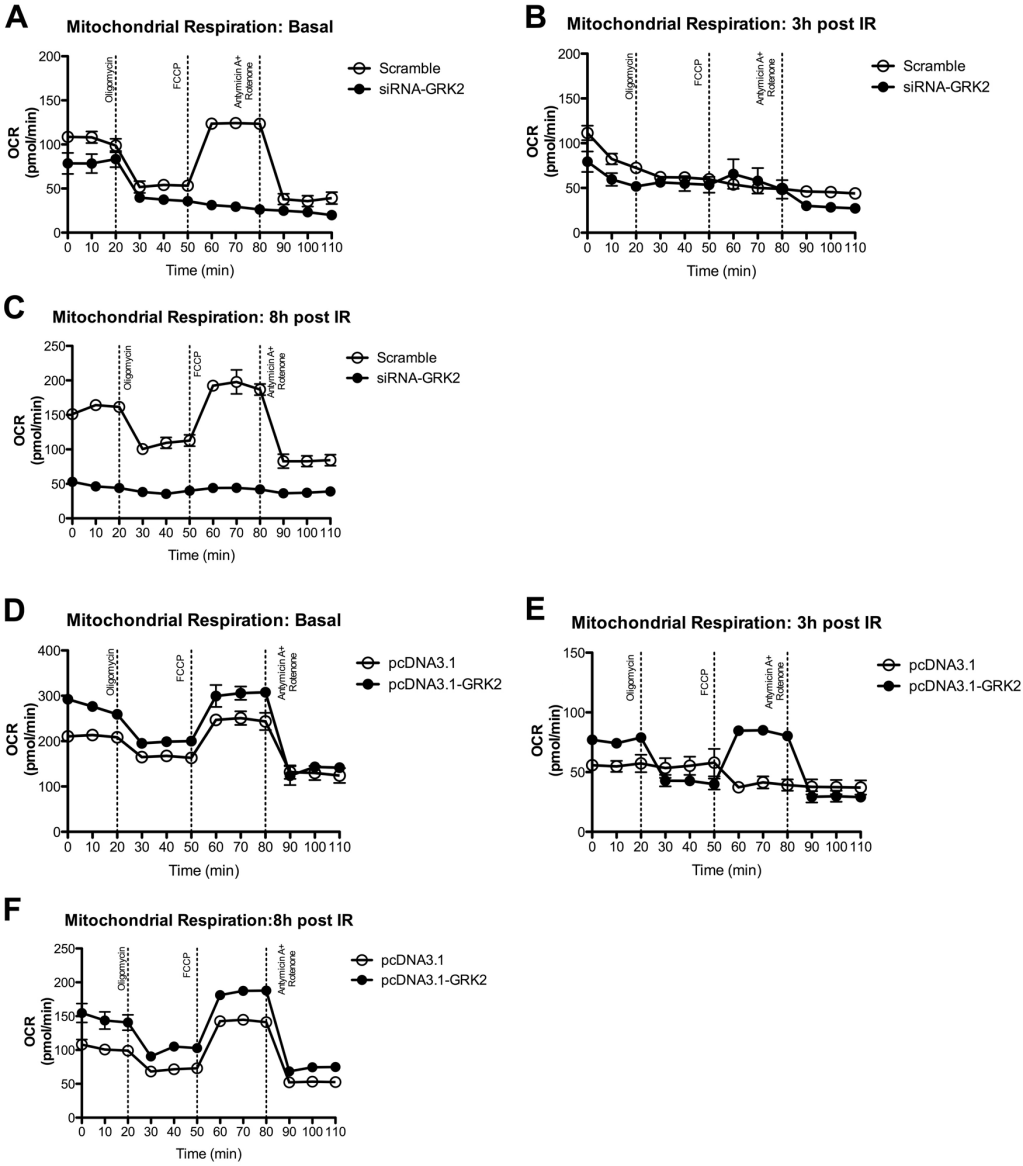
Change of GRK2 intracellular levels affects mitochondrial respiration after IR exposure. **a–c** GRK2 silencing impairs mitochondrial respiration and recovering after IR exposure in HEK293. Representative graphs of *n* = 2 independent experiments of HEK293 cells treated with scramble-siRNA and siRNA-GRK2 in basal conditions, at 3 and 8 h post IR exposure. HEK293 cells treated with siRNA-GRK2 have a compromised OCR already in basal condition compared to control cells (**a**). IR exposure blunts mitochondrial respiration at 3 h in both conditions (**b**) but with no recovery at 8 h post-IR in cells with GRK2 silencing (**c**).** d**–**f**. GRK2 preserves mitochondrial respiration and facilitates recovering after IR exposure in HEK293. Representative graphs of *n* = 2 independent experiments of HEK293 cells treated with pcDNA3.1 and pcDNA3.1-GRK2 at basal, 3 h and 8 h post IR. GRK2 overexpression enhances mitochondrial respiration at basal condition (**d**) and preserves mitochondrial respiration at 3 h post-IR (**e**). Full recovery of mitochondrial respiration at 8 h post-IR in both conditions is enhanced by GRK2 overexpression as compared to control cells (**f**)

To assess the impact of GRK2 silencing or overexpression on mitochondrial function, we measured mitochondrial respiration by determining the cellular oxygen consumption rate (OCR) in HEK-293 cell treated with siRNA-GRK2 or pcDNA3.1-GRK2, in which GRK2 has been silenced or overexpressed, respectively. Figure 3a describes the different pattern of OCR in HEK-293 treated with scramble-siRNA and siRNA-GRK2 in basal condition, in the absence of irradiation. HEK293 treated with scramble displays a typical OCR profile in response to the different mitochondrial complex inhibitors. HEK-293 cells treated with siRNA-GRK2, instead show a compromised OCR response to the mitochondrial inhibitors. IR exposure affects mitochondrial respiration in both scramble and siRNA-GRK2 treated cells at 3 h (Fig. 3b). At 8 h post IR exposure, HEK-293 cells in which GRK2 is silenced, do not recover mitochondrial function and respiration, differently from what observed in the cells treated with the scramble (Fig. 3c).

Next, we analyzed the effects of GRK2 overexpression on the bioenergetic profile of HEK-293 cells (Fig. 3d–f). GRK2 overexpression enhances mitochondrial respiration as compared to control cells treated with pcDNA3.1(Fig. 3d). At 3 h post IR exposure, mitochondrial respiration is compromised in control cells, while in HEK-293 overexpressing GRK2 the response to the mitochondrial complex inhibitors was partly preserved, although the OCR profile was reduced respect to that observed in non-irradiated cells (Fig. 3e). Finally, at 8 h post IR exposure, mitochondrial respiration is fully recovered in both control and GRK2 overexpressing cells (Fig. 3f)

### GRK2 dynamically interacts with MFN-1 after IR exposure

**Fig. 4:**
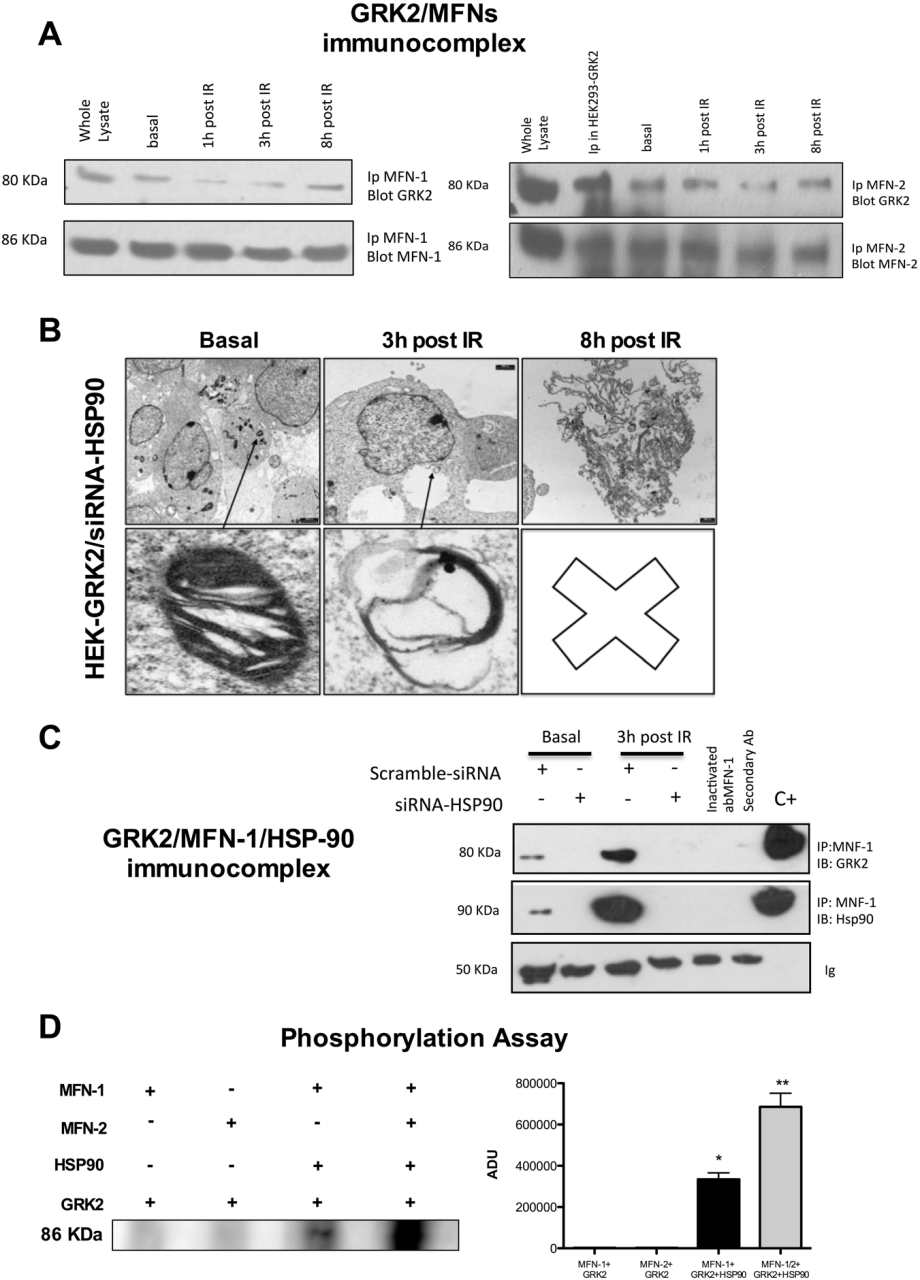
GRK2 interacts and phosphorylates MFNs. **a** GRK2 dynamically interacts with MFNs after IR exposure in HEK293 cells. Immunoprecipitation assay performed on the whole lysate of HEK293 cells exposed to IR. Representative image of *n* = 3 independent experiments. Whole lysate from HEK293 cells and IP in HEK293-GRK2 cells represent positive control. GRK2 interacts with MFN-1 and 2 already in basal condition. IR exposure reduces GRK2/MFNs complex with the following recovery at 8 h post-IR. **b** HSP90 silencing removes protective effects of GRK2 overexpression on mitochondrial morphology after IR exposure*.* Representative TEM image of HEK293 with stable GRK2 overexpression and treated with siRNA for HSP90 (*n* = 3 independent experiments). As shown above, GRK2 overexpression enhances mitochondrial recovering after IR exposure (Fig. 2). In siRNA-HSP90 treated cells, recover from mitochondrial morphology and number at 8 h post-IR cannot be observed. **c** The GRK2/MFN1 complex formation is HSP90 dependent*.* Immunoprecipitation assay performed on the whole lysate of HEK293 cells with stable GRK2 overexpression treated with scramble or siRNA for HSP90. Representative image of *n* = 3 independent experiments. Ip with secondary Ab and inactivated MFN1ab are negative control. Whole lysate represents the positive control. IR exposure increases GRK2 interaction at 3 h post-IR. HSP90 removal abolishes GRK2/MFN1 interaction. **d** GRK2 phosphorylates MFN1/2 in the presence of HSP90. In vitro kinase assay with purified MFN1/2, HSP90, and GRK2. Representative image of *n* = 3 independent experiments. GRK2 alone does not produce phosphorylation of MFNs. GRK2 induces MFN-1 phosphorylation when HSP90 is added into the assay (* vs. MFN-1 + GRK2, *p* < 0.05). The presence of both MFNs produces a cumulative effect (** vs. MFN1/2 + GRK2; *p* < 0.05)

The above data let hypothesize a role for GRK2 to regulate the complex molecular machinery involved in the mitochondria homeostasis. In particular, we focused on the process of fission and fusion, since these opposing forces dynamically regulate mitochondrial shape, ultrastructure, and function and are reciprocally inhibited and activated by a wide range of stressful events^16–18^. We found that GRK2 dynamically interacts with MFNs. As shown in Fig. 4a GRK2/MFN-1 interaction is reduced at 1 h and 3 h but recovered at 8 h post-IR exposure. Similarly, GRK2 interaction with MFN2 is significantly reduced at 3 h and then retrieved at 8 h post-IR. Stress proteins mediate acute cellular responses, and GRK2 is delivered into mitochondria through binding with HSP90 [5]. In HEK-293 with stable overexpression of GRK2 (HEK-GRK2), HSP90 silencing affects mitochondrial morphology already in basal condition and removes the protective effects of GRK2 overexpression (Fig. 4b) as shown in Fig. 2. Therefore, we evaluated the MFN-1/GRK2 interaction after HSP90 silencing by siRNA. In HEK-GRK2, the interaction with MFN-1 is enhanced after IR exposure. HSP90 silencing abolishes interaction of GRK2 with MFN-1 (Fig. 4c), indicating the critical role of this chaperone in assembling GRK2/MFN-1 complex. Next, to investigate whether the complex MFN1/GRK2/HSP90 leads to MFN1 phosphorylation, we performed an in vitro ^32^P labeling assay with purified proteins. As shown in Fig. 4c, GRK2 phosphorylates MFN-1 only in the presence of HSP90. Cumulative effects are observed when both MFNs are added in the assay (Fig. 4d).

## Discussion

This work identifies for the first time that MFN1 and 2 are the molecular partners of GRK2 in mitochondria. MFNs share N-T (GTPase) domain, two coiled-coil heptad repeat regions (HR-1 and HR-2), and two trans-mitochondrial membrane domains^19^, regulated by a series of post-translational modifications^20^. Moreover, in recent studies, it has been established that MFN-1-2 are in basal condition prevalently in non-tethering and no fusion conformation, with HR2 and HR1 domains folded with anti-parallel bonds^21^. Phosphorylation of these domains promotes desensitization of the bonds, and it allows the opening and extension into the cytosol of HR-2, in a tethering-permissive and pro-mitochondrial fusion state^15^. In the current work, we found that GRK2 dynamically binds and phosphorylates MFN-1/2 (Fig. 4a–c) and this may allow the opening of the HR1/HR2 domain with following mitochondrial tethering and fusion. The GRK2/HSP90/MFN1-2 is a previously unknown pathway involved in cellular pathophysiology that, we believe, can have significant implications for the prevention or treatment of the organ damage following IR exposure or other stress conditions. Our finding is also the first report of a kinase regulating the MFN1-2 activity. Other kinases might as well be able to phosphorylate MFN1-2, and in this sense, our report opens a new scenario of tight regulation of mitochondrial responses to transmembrane signal transduction.

The above data profoundly change our understanding of the cellular role of GRK2, showing for the kinase the role of a stress protein able to compartmentalize in mitochondria upon an acute and stressful event, where it accomplishes pro-regenerative and protective functions on mitochondria.

According to these results, we believe that the role of GRK2 in cell physiology or disease needs to be redefined, in particular distinguishing from acute and chronic conditions. A consolidated body of evidence shows that on the long-term (months or years), excessive levels of GRK2 are deleterious^22,23^, and its inhibition and modulation by molecular or pharmacological strategy have beneficial effects on the outcome of animal models of HF^24,25^. On the other side, GRK2 is a central signaling node through which several signaling pathways converge to regulate cellular functions and homeostasis^1^. Thus, it is not surprising that lack of GRK2 is deleterious for embryonic cardiovascular development^26^ or that in the adult life, cardiac-selective GRK2 removal leads to an eccentric dilatation of the heart^27^. Moreover, endothelial-selective GRK2 removal induces early atherosclerosis in the aortas and inhibits neoangiogenesis in mice^28,29^. The current study follows this groove, showing that in an acute condition (hours), like after IR exposure, GRK2 overexpression is protective, while its removal abolishes any possibility for mitochondrial and cell to recover after the stress evoked by IR exposure. Thus, while an excessive GRK2 activity appears deleterious for the cell in chronic conditions, a proper and rapid mobilization of this molecule after the acute stress is needed for the cell survival.

## Material and methods

### Cell culture

HEK-293 cells were cultured within Dulbecco’s minimal essential medium (DMEM) and 25 mM glucose supplemented with 10% fetal bovine serum (FBS) at 37 °C in 95% air and 5% CO2. HEK-293 Cells are plated, and exposed single dose of 4 Gray (4Gy) X-radiation at room temperature in medium with FBS (250 KV, 16Ma, Dose Rate: 1.5 Gy/Min) measurements were performed from 3 and 8 h after the exposition.

### Cell transfection

Cell transfection was performed with lipo transfection reagent (Lipofectamine 2000, Invitrogen) using 2 µg of plasmid DNA according to manufacturer’s instructions. For stably transfected clone selections, after 24 to 48 h cell medium was supplemented with G418, (500 µg/ml) for two weeks. The G418-resistant cell was cultured in medium supplemented with G418 to a final concentration of 250 µg/ml and examined for overexpression of GKR2 (HEK-293 GRK2) by western blot, using a specific antibody, (SantaCruz Biotechnology).

### siRNA transfection

Small interfering RNA (siRNA) for GRK2 and Scramble used as the control for all siRNA experiments (IDT Integrated DNA Technologies). (siRNA GRK2:5′-CUCCAGCUUCUCGUAUUUCUUTT-3′ Scramble GRK2:5′-UGUCGAUCUCAUGCUUGUCUUTT-3′). siRNA HSP90 α/β.

The siRNAs were transfected according to manufacturer’s instructions. After 48 to 72 h, the cells were examined for Knock down of HSP 90 α/β and GRK2 by Western Blot using specific antibody (SantaCruz Biotechnology).

### Immunoprecipitate and Western Blot

Cells were lysed in RIPA/SDS buffer [50 mM Tris-HCl (pH 7.5), 150 mM NaCl, 1% Nonidet P-40, 0, 25% deoxycholate, 9,4 mg/50 ml sodium orthovanadate, 20% SDS]. Protein concentration was determined by using BCA assay kit (Pierce). Endogenous MNF1-2 from total extracts were immunoprecipitated with specific antibodies (Santa Cruz) and protein A/G agarose (Santa Cruz). After extensive washing, the immunocomplexes were electrophoresed by SDS/PAGE and transferred to nitrocellulose; Mnf 1-2, HSP90α/β, or GRK2 were visualized by specific antibody (Santa Cruz), anti-mouse HRP-conjugated secondary antibody (Santa Cruz) and standard chemiluminescence (Pierce). The whole lysate was used as positive control. As the negative controls, the assay was performed using a nonspecific antibody from the same species as the IP antibody and heated and inactivated MFN-1 (90 °C for 1 min).

For western blot analysis, cells were lysed in ice-cold RIPA/SDS buffer [50 mmol/L Tris-HCl (pH 7.5), 150 mmol/L NaCl, 0.01 g/L NP-40, 0.0025 g/L deoxycholate, 2 mmol/L Na_3_VO_4_, 0.2 g/L sodium dodecyl sulfate and Protease Inhibitor cocktail (SIGMA)]. Protein concentration was determined using BCA assay kit (Pierce), equal amounts of total cellular extracts were electrophoresed on 4–12% SDS-PAGE gel and transferred to the Immobilon-P nitrocellulose filter (Millipore Corporation). The membranes were blocked in Tris-buffered saline containing 0.002 g/L Tween 20 (TBST) and 0.05 g/L nonfat dry milk. After blocking, the membranes were washed three times with TBST and then incubated overnight at 4 °C in TBST containing 5% BSA with primary specific antibody: The antibodies anti-Mnf 1-2, actin, and GRK2 were from Santa Cruz Biotechnology, Inc. Blots from 3 independent experiments were quantified and corrected for appropriate loading control. Densitometric analysis was performed using Image Quant software (Molecular Dynamics, Inc). Results are reported as mean ± SEM.

### Mitochondria extracts

Cells were washed in ice-cold phosphate buffer (PBS) and disrupted by Dounce homogenization in isolation buffer [IB pH 7.4 200 mM sucrose, 1 mM EGTA-Tris and 10 mM Tris-MOPS]. The homogenate was spun at 800×*g* for 10 min; the supernatant was recovered and further centrifuged for 10 min at 8000×*g*. The resulting pellet (mitochondrial fraction) was collected while the supernatant was further spun for 30 min at 100,00×*g* to obtain the cytosolic fraction, spanned again at 100,000×*g* to further purify the fraction. The mitochondrial fraction was further purified by centrifuging twice at 8000×*g* for 10 min. The obtained pellet was clarified by centrifugation at 95,000×*g* for 30 min on a 30% Percoll gradient in IB. The obtained mitochondrial layer was washed free of Percoll and resuspended in IB. Protein concentration was determined by bicinchoninate assay (Pierce). Cytosol and mitochondrial extracts were confirmed by western blot, as previously described using specific antibodies (Santa Cruz Biotechnology). Densitometric analysis was performed using Image Quant software (Molecular Dynamics, Inc). Results are reported as mean ± SEM.

### Plasma membranes extracts

For plasma membrane isolation, cells were homogenized in buffer containing 25 mM Tris-HCl (pH 7.5), 5 mM EDTA, 5 mM EGTA, 1 mM phenylmethylsulfonyl fluoride, 2 μg/ml each leupeptin and aprotinin. In experiments that required detection of phosphoproteins lysis buffers also contained phosphatase inhibitors (Sigma). Intact cells and nuclei were moved by centrifugation at 1000×*g* for 15 min. The collected supernatant was further subject to centrifugation at 38,000×*g* for 1 h. The pellet was resuspended in lysis buffer (1% Nonidet P-40, 10%glycerol, 137 mM NaCl, 20 mM Tris-HCl (pH 7.4), 1 mM phenylmethylsulfonyl fluoride, 20 mM NaF, 1 mM sodium pyrophosphate, 1 mM sodium orthovanadate, and 2 μg/ml each aprotinin and leupeptin) and used as plasma membrane fraction, and the supernatant was used as the cytosolic fraction. Cytosolic fractions and membrane fraction were confirmed by western blot, as previously described using specific antibodies for GRK2 (SantaCruz). Densitometric analysis was performed using Image Quant software (Molecular Dynamics, Inc). Results are reported as mean ± SEM.

### Cytofluorimetry

Cells were incubated 37 °C, 95% air and 5% of CO2, for 30 min with 5 nM Mito Tracker (Rosamine-based Mitotracker dye-Invitrogen) to identify mitochondria, 5 μ M (Red Mitochondrial Superoxide Indicator) MitoSOX to determine Ros mitochondria production, 100 n M TMRE (Tetramethylrhodamine, Ethyl, Ester) to identify mitochondrial potential membrane. Then Mito tracker, Mitosox, and TMRE stained and unstained control cells were analyzed by flow cytometry (FACS Calibur, BD Biosciences) followed by analysis of mean fluorescence intensity of 10,000 events by Cellquest software (BD Biosciences).

### Metabolism assay

Real-time measurements of oxygen consumption rate (OCR) was made using an XF-96 Extracellular Flux Analyzer (Seahorse Bioscience). Cells were plated in XF-96 plates (Seahorse Bioscience) at the concentration of 20,000 cells/well. OCR was measured in XF media (non-buffered DMEM medium, containing 10 mM glucose, 2 mM L-glutamine, and 1 mM sodium pyruvate), under basal conditions and in response to 5 μM oligomycin, 1.5 μM of carbonyl cyanide-4- (trifluoromethoxy)-phenylhydrazone (FCCP) and 1 μM of Antimycin and Rotenone (all from Sigma-Aldrich). OCR was measured in 2 separate experiments with at least *N* = 3 replicated per sample. Representative OCR graphs are shown in Fig. 3.

### Transmission electron microscopy

Cells with overexpression and deletion for GRK2 and HSP90 deletion were fixed with 2.5% glutaraldehyde, washed in PBS, fixed in osmium tetroxide, dehydrated in an ethanol series, embedded in epoxy resin, and then examined under a transmission electron microscope (JEM-2000EX), at the Federico II facility for advanced imaging (CISME). The mitochondrial aspect ratio (the ratio of length/width) was calculated using ImageJ Software.

### Kinase activity assay

To investigate in vitro phosphorylation of MNF-1/2 by GRK2, 50 ng of active GRK2 were assayed on 100 ng of purified Mnf ½. Phosphorylation reactions were initiated by adding 20 mM ATP, 1 mM CaCl2, 20 mM MgCl2, 4 mM Tris, pH 7.5, and 10 Ci of [32 P]γ‐ATP (specific activity 3000 Ci/mmol) and prolonged for 30 min at 37 °C. Laemmli buffer was added to stop the reaction. Then, 30 µl of the reaction mix were resolved on SDS‐ PAGE 4‐12% gradient (Invitrogen), stained with Coomassie blue, destained, vacuum dried and exposed for autoradiography.

### Statistical analysis

All values are presented as mean ± SEM. Two-way ANOVA was performed to compare the different parameters among the different groups. Bonferroni post hoc testing was performed where applicable. A significance level of *p* < 0.05 was assumed for all statistical evaluations. Statistics were computed with GraphPad Prism software (San Diego, California).

## Electronic supplementary material

supplemental figure 1
