# Supplementary material for: GRK2 moderates the acute mitochondrial damage to ionizing radiation exposure by promoting mitochondrial fission/fusion
Source: Cell Death Discov. 2018 Feb 14;4:25. doi: 10.1038/s41420-018-0028-7 (PMC5841414; doi:10.1038/s41420-018-0028-7)
Supplement: Supplementary file 1 — supplemental figure 1 [file 41420_2018_28_MOESM1_ESM.pdf]

Supplemental Figure 1

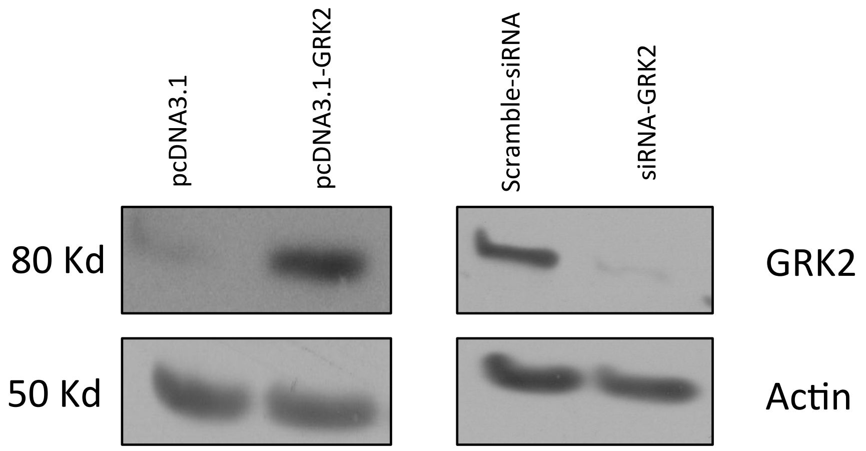

Western Blot performed on the whole lysate of HEK293 cells transfected with pcDNA3.1 or siRNA-GRK2. Representative image showing GRK2 overexpression and silencing.
